# Observing Huntington’s Disease: the European Huntington’s Disease Network’s REGISTRY

**DOI:** 10.1371/currents.RRN1184

**Published:** 2011-04-13

**Authors:** Michael Orth, Olivia J Handley, Carsten Schwenke, Stephen B. Dunnett, David Craufurd, Aileen K Ho, Edward Wild, Sarah J Tabrizi, G. Bernhard Landwehrmeyer, the investigators of the European Huntington's Disease Network, Raphael M. Bonelli, Brigitte Herranhof, Anna Hödl, Michael Koppitz, Markus Magnet, Daniela Otti, Annamaria Painold, Karin Reisinger, Anja Flamez, Vera Morez, Sylvie de Raedt, Pascale Ribaï, Christine Verellen-Dumoulin, Wim Vandenberghe, Dimphna van Reijen, Lis Hasholt, Lena E. Hjermind, Oda Jakobsen, Anne Nørremølle, Sven Asger Sørensen, Jette Stokholm, Maarit Peippo, Marjatta Sipponen, Heli Hiivola, Kirsti Martikainen, Katri Tuuha, Christoph Michael Kosinski, Daniela Probst, Christian Sass, Johannes Schiefer, Christiane Schlangen, Cornelius J. Werner, Josef Priller, Harald Prüß, Jürgen Andrich, Rainer Hoffmann, Peter Kraus, Christian Prehn, Carsten Saft, Stephan Salmen, Katrin Straßburger, Herwig Lange, Ulrike Hunger, Matthias Löhle, Simone Schmidt, Alexander Storch, Anett Wolz, Martin Wolz, Johann Lammbeck, Birgit Zucker, Ute Hidding, Alexander Münchau, Michael Orth, Lars Stubbe, Walburgis Heinicke, Michael Orth, Bernhard Longinus, Jens Carsten Möller, Ida Rissling, Alexander Peinemann, Michael Städtler, Adolf Weindl, Stefan Bohlen, Herwig Lange, Ralf Reilmann, Antonie Beister, Matthias Dose, Gabriele Leythaeuser, Ralf Marquard, Caroline Schrenk, Michele Schuierer, Alexandra Wiedemann, Daniel Ecker, Bernhard Landwehrmeyer, Franziska Lezius, Sonja Trautmann, Elisabetta Bertini, Claudia Mechi, Marco Paganini, Sivia Piacentini, Maria Romoli, Sandro Sorbi, Giovanni Abbruzzese, Monica Bandettini di Poggio, Emilio Di Maria, Giovanna Ferrandes, Paola Mandich, Roberta Marchese, Alberto Albanese, Stefano Di Donato, Caterina Mariotti, Paola Soliveri, Rinaldi Carlo, Di Maio Luigi, Giuseppe De Michele, Carlo Rinaldi, Elena Salvatore, Tecla Tucci, Andrea Ciarmiello, Tiziana Martino, Maria Simonelli, Ferdinando Squitieri, Anna Rita Bentivoglio, Alfonso Fasano, Marina Frontali, Arianna Guidubaldi, Tamara Ialongo, Gioia Jacopini, Giovanna Loria, Carla Piano, Silvia Romano, Francesco Soleti, Maria Spadaro, Paola Zinzi, Arvid Heiberg, Marleen R van Walsem, Kathrine Bjørgo, Madelein Fannemel, Per Gørvell. Lars Retterstøl, Inga Bjørnevoll, Sigrid Botne Sando, Emilia Jadwiga Sitek, Jaroslaw Slawek, Witold Soltan, Magdalena Boczarska-Jedynak, Barbara Jasinska-Myga, Gregorz Opala, Monika Rudzińska, Andrzej Szczudlik, Magdalena Wójcik, Krzysztof Banaszkiewicz, Anna Bryl, Anna Ciesielska, Aneta Klimberg, Wojciech Kozubski, Jerzy Marcinkowski, Pani Justyna Sempołowicz, Daniel Zielonka, Piotr Janik, Anna Kalbarczyk, Hubert Kwiecinski, Zygmunt Jamrozik, Jakub Antczak, Grzegorz Witkowski, Maryla Rakowicz, Przemyslaw Richter, Danuta Ryglewicz, Jacek Zaremba, Elzbieta Zdzienicka, Christina Costa, Miguel Coelho, Joaquim J Ferreira, Tiago Mestre, Mário M Rosa, Anabela Valadas, Miguel Gago, Carolina Garrett, Maria Rosalia Guerra, Jordi Bas, Matilde Calopa, Miquel Aguilar Barberà, Dolores Badenes, Laura Casas, Sonia Escalante Arroyo, Jorge Hernández Vara, Jerzy Krupinski, Judith López, Marta Obdulia, Pilar Quilez Ferrer, Ana Rojo Sebastián, Silvia Romero Contreras, Gemma Tome Carruesco, Esther Cubo, Natividad Mariscal, Jesús Sánchez, Francisco J Barrero, Blas Morales, José Luis López-Sendón Moreno, Rocío García-Ramos García, Purificacion Pin Quiroga, Clara Villanueva, Pedro-José García Ruíz-Espiga, Asunción Martínez, María José Saiz Artiga, Vicenta Sánchez, Mónica Bascuñana, Marta Fatas, Guillermo García Ribas, Justo García de Yébenes, José Luis López Moreno, Christine Schwarz, Patricia Trigo Cubillo, Penelope Navas Arques, Aranzazú Gorospe, Inés Legarda, María José Torres Rodríguez, Itziar Gaston, Maria A. Ramos-Arroyo, Javier López del Val, Laura Martinez, Jean-Marc Burgunder, Irene Romero, Michael Schüpbach, Sabine Weber Zaugg, Monique S.E. van Hout, Jeroen P.P. van Vugt, A. Marit de Weert, J.J.W. Bolwijn, Meike Dekker, K.L. Leenders, Joost.C.H. van Oostrom, Reineke Bos, Eve Dumas, Caroline K. Jurgens, Raymund A.C. Roos, Marie-Noëlle Witjes-Ané, Kirsty Matheson, Daniela Rae, Sheila Simpson, Fiona Summers, Alexandra Ure, Adrienne Curtis, Jenny Keylock, Hugh Rickards, Jan Wright, Roger A. Barker, Kate Fisher, Anna Olivia Goyder Goodman, Susan Hill, Ann Kershaw, Sarah Mason, Nicole Paterson, Lucy Raymond, Jon Bisson, Monica Busse, Lynda Ellison-Rose, Olivia Handley, SB Dunnett, Jenny Naji, Kathy Price, Anne Rosser, Maureen Edwards, Paul A. De Sousa, Teresa Hughes, Marie McGill, Pauline Pearson, Mary Porteous, Adam Zema, Peter Brockie, Jillian Foster, Nicola Johns, Sue McKenzie, Gareth Thomas, Liz Burrows, Amy Fletcher, Fiona Laver, Mark Silva, Aileen Thomson, Carol Chu, Emma Hobson, Stuart Jamieson, Jean Toscano, Sue Wild, Pam Yardumian, Colin Bourne, Carole Clayton, Heather Dipple, Janet Grant, Diana Gross, Caroline Hallam, Julia Middleton, Ann Murch, Thomasin Andrews, Andrew Dougherty, Fred Kavalier, Charlotte Golding, Alison Lashwood, Dene Robertson, Deborah Ruddy, Anna Whaite, Thomasin Andrews, Stefania Bruno, Charlotte Golding, Susie Henley, Marianne Novak, Christine O'Driscoll, Aakta Patel, Elisabeth Rosser, Sarah Tabrizi, Rachel Taylor, Thomas Warner, Edward Wild, Natalie Arran, David Craufurd, Ruth Fullam, Liz Howard, Susan Huson, Lucy Partington-Jones, Nichola Ritchie, Julie Snowden, Annie Solom, Cheryl Stopford, Jennifer Thompson, Leann Westmoreland, Andrea H Nemeth, Gill Siuda, Oliver Bandmann, Alyson Bradbury, Kay Fillingham, Isabella Foustanos, Oliver Quarrell, Hazel Reynders, Lisa Robertson, Katharine Tidswell

**Affiliations:** ^*^Department of Neurology, University of Ulm, Ulm Germany; ^†^Department of Neurodegenerative Disease, UCL Institute of Neurology, London, UK; ^‡^Scossis, Statistics and Consulting, Berlin, Germany; ^§^The Brain Repair Group, Cardiff University, Cardiff, Wales, UK; ^¶^University of Manchester, Manchester Academic Health Sciences Centre and Central Manchester University Hospitals NHS Foundation Trust, Manchester, UK; ^#^University of Reading, School of Psychology, 3 Earley Gate, WhiteKnights Road, Reading, RG6 6AL and ^**^UCL Institute of Neurology, London, UK; Bristol University, UK

## Abstract

Background: Huntington’s disease (HD) is a rare triplet repeat (CAG) disorder. Advanced, multi-centre, multi-national research frameworks are needed to study simultaneously multiple complementary aspects of HD. This includes the natural history of HD, its management and the collection of clinical information and biosamples for research.

Methods: We report on cross-sectional data of the first 1766 participants in REGISTRY, the European Huntington’s Disease Network’s (EHDN), multi-lingual, multi-national prospective observational study of HD in Europe. Data collection (demographics, phenotype, genotype, medication, co-morbidities, biosamples) followed a standard protocol.

Results: Phenotype, and the HD genotype, of manifest HD participants across different European regions was similar. Motor onset was most common (48%) with a non-motor onset in more than a third of participants. Motor signs increased, and cognitive abilities and functional capacity declined as the disease burden (CAGn-35.5) X age) increased. A life-time history of behavioural symptoms was common, but the behavioural score was not related to disease burden. One fifth of participants had severe psychiatric problems, e.g. suicidal ideation and attempts, and/or irritability/aggression, with psychosis being less common. Participants on anti-dyskinetic medication had a higher motor and lower cognitive score, were older, and more prone to physical trauma. A higher motor and a lower cognitive score predicted more advanced disease.

Conclusions: The unparalleled collection of clinical data and biomaterials within the EHDN’s REGISTRY can expedite the search for disease modifiers (genetic and environmental) of age at onset and disease progression that could be harnessed for the development of novel treatments.

I**ntroduction**

Huntington’s disease (HD) is an autosomal dominant neurodegenerative disorder caused by a CAG repeat expansion in the *HTT* gene. HD usually manifests in adult life, causing motor impairments, cognitive decline and behavioural/psychiatric alterations [1] . HD is devastating and inevitably fatal; currently, no disease-modifying treatment is established [2] .

Historically, the study of HD has benefited strikingly from multi-centre research initiatives, typified by the international collaborative effort that identified the causative CAG repeat expansion in the *HTT* gene in 1993 [3]. Rapid advances in molecular and cellular biology and genetics have produced a wealth of insights at the molecular level [4] . Much effort at present is focused on identifying therapeutic targets and developing treatments that may delay onset of the disease, or slow down or stop the progression of HD once it manifests. 

With a prevalence of 5-8/100 000, manifest HD is relatively rare. Thus we need advanced, multi-centre, multi-national research frameworks that allow us to study simultaneously multiple complementary aspects of HD. This includes the natural history of HD, its management and the collection of clinical information and biosamples for research. The European Huntington’s Disease Network (EHDN; www.euro-hd.net), established in 2004, is a collaborative network of HD researchers, HD clinicians, people affected by HD, and their relatives across 18 European countries. It strives to lay the foundations on which to advance knowledge about HD, how to optimally assess disease progression and factors that modify the phenotype. This initiative aims to develop new symptomatic therapies, and provide the infrastructure to test rapidly putative disease-modifying treatments in a multi-centre, multi-national setting with the ultimate goal of improving the quality of life of people affected by the disease [5] .

In the present paper we report on cross-sectional enrolment data of a first cohort of participants in REGISTRY, EHDN’s core observational study. REGISTRY is a multi-centre, prospective observational study with annual follow-up visits that enrols manifest and pre-manifest HD expansion mutation carriers, individuals at risk of HD, non-mutation gene carriers, and controls (no family history of HD). In the present study, we assessed the HD genotype and phenotype across different European regions. We evaluated the phenotype and its variability in relation to CAG repeat length and age as key biological factors; and European region, treatment modality and co-morbidity as environmental factors. Finally, we examined potential predictors of functional capacity in domains relevant to daily life.

**Methods and materials**

*Participants*

This report is based on monitored data from the enrolment visit of the first 1766 participants (98% Caucasians) in 66 study sites from 13 European countries within the European Huntington’s Disease Network (EHDN). 

Participants gave informed written consent according to the International Conference on Harmonisation-Good Clinical Practice (ICH-GCP) guidelines (http://www.ich.org/LOB/media/MEDIA482.pdf). For participants who lacked capacity to consent study sites adhered to country-specific guidelines for obtaining consent. Minors assented with both parents consenting for them. Ethical approval was obtained from the local ethics committee for each study site contributing to REGISTRY.

*Study design*

Data collection followed a standard protocol (Table 1) using electronic case report forms available in Czech, Danish, Dutch, English, Finnish, French, German, Italian, Norwegian, Polish, Portuguese, Spanish, and Swedish. At each centre, clinicians with long standing experience in HD took a careful history and examined patients clinically; motor, psychiatric and cognitive signs were scored using the Unified Huntington’s Disease Rating Scale (UHDRS) [6] . Assessments were complemented by self-rating scales that probed mood, quality of life and health economics (for an overview and references see Table 1). Disease stage was derived from the Total Functional Capacity (TFC) scores [7] .

**Table 1.** The complete REGISTRY assessment protocol

**Table:** 

General	Medical History (medical, disease, psychiatric)
	Demographics (Fixed & Variable)
	Comorbid conditions
	Concomitant medication
	Family History
	CAG
Clinical assessment	UHDRS '99 Motor, TFC, Functional [6]
	UHDRS '99 Behaviour [6]
	Becks Depression Inventory [8]
	Hamilton Depression Rating Scale [9]
Cognitive assessments	UHDRS '99 Cognitive (verbal fluency, symbol digit modality test, colour naming, word reading, interference) [6]
Quality of Life	SF-36 [10] Caregiver Burden Inventory [11]
Health economics	Client Service Receipt Inventory [12]
Biosample collection	30 ml blood, 30 ml urine

All participants were assigned a 9-digit pseudonym created using a secure one-way hash algorithm. No identifying data were stored on the EHDN server. Data was entered on-line using an electronic web-based data capture system (www.euro-hd.net) where a username determines access rights within the web portal. No identifying data were stored on the EHDN server. Entries for medication were coded according to the Anatomical Therapeutic Chemical (ATC) classification (www.whocc.no/atcddd), and co-morbidities were coded according to ICD-10. Data entry onto the webportal was subject to automatic plausibility checks. In addition, study site raters were annually trained, assessed and certified to reduce inter- and intra-rater variability. Following data entry data were monitored on-line and on-site by monitors fluent in the language of the contributing study site. Data monitoring adhered to the principles laid out in ICH-GCP.  

*Biosample collection*

Blood was collected and shipped to BioRep at room temperature for genetic analysis and lymphoblastoid cell line creation (BioRep, Milan, Italy) [13] [14]. DNA was extracted [15], and *HTT* gene CAG repeat length was analysed (PCR amplification followed by capillary electrophoresis using the MegaBace Fragment Profiler Software from General Electric, Buckinghamshire, UK [16] [17]. A second, independent, accredited laboratory in Tübingen, Germany, duplicated CAG repeat analyses (Applied Biosystems, CA, USA). Mid-stream urine samples were collected for biomarker studies. DNA and urine were stored at -80°C.

*Data analyses*

Descriptive statistics were calculated for quantitative variables and frequency counts by category for qualitative variables. Confidence intervals were calculated where appropriate. If not stated otherwise, these intervals were two-sided and provided 95% confidence. For qualitative variables Chi² tests or Fisher's exact tests were used, for quantitative variables t-tests, Kruskal-Wallis tests (global) and Wilcoxon-rank-sum-tests (pairwise comparisons) were used as appropriate. All tests were performed two-sided where p-values below 5% were regarded as statistically significant. 

Cohen's kappa examined the agreement of the laboratories measuring the CAG repeat lengths [18] .

Linear regression analysis with F-test examined which factors influence continuous variables. Logistic regression evaluated factors explaining absence/presence of suicidal attempts. Multinomial logistic regression was performed to select factors important for the prediction of the disease stage in this cross-sectional analysis and to perform receiver operating characteristic (ROC) analysis to evaluate the predictive value of important factors for the disease stage. 

Calculations were performed using R software (version 2.7.1, R Development Core Team (2008)) and NCSS 2007 (NCSS, Kaysville, Utah, USA). 

**Results**

*Participants and genotype*

Of 1766 participants, 1540 had manifest HD, 226 were pre-manifest gene mutation carriers defined as carrying the HD gene mutation and having a diagnostic confidence score of less than 4 on the UHDRS motor scale [6] . The median estimated age at onset in manifest HD participants was 43 years (range 10-87 years). In 795 patients (45%) the gene was inherited from the mother and in 710 (40.2%) from the father. In 25 participants (1.5%), there was no known family history, and in 234 (13.3%) the information was missing. Thirty-two participants (2.1%) had a juvenile-onset (before the age of 20, [19]), and 96 (5.4%) had a late-onset of HD (above the age of 60). 

*Clinical phenotype*

We analysed 1468 participants from 13 countries where a complete set of demographic data (age, sex), CAG repeat length (large allele), UHDRS motor score and TFC were all available (Table 2). Numbers of participants from individual countries were too low for meaningful statistical analyses. For this reason we arbitrarily collapsed participants by geographical region. 514 were from Central Europe (Germany, Austria, Switzerland, Netherlands, Belgium), 110 were from Northern Europe (Denmark, Norway, Finland), 457 were from Southern Europe (Italy, Spain, Portugal), 334 were from the United Kingdom, and 53 were from Poland. 784 were female. 1280 had manifest HD, and 188 had premanifest HD (Table 2).

**Table 2. ** Clinical and genetic data from REGISTRY participants

**Table:** 

** **	**All**	**Central European^1^**	**Northern European^2^**	**Southern European^3^**	**UK**
**Age (median, range)**	49 (10-93)	47 (20-87)	50 (23-93)	49 (11-83)	49 (17-85)
**Male:female**	0.87	0.85	1.08	0.92	0.79
**CAG (median, range)**	44 (36-90)	43 (38-65)	42 (36-51)	44 (36-90)	43 (37-85)
**Reduced penetrance range (%)**	45 (3.1)	9 (1.8)	11 (10)	10 (2.2)	12 (3.6)
**Premanifest (% total)**	188 (12.8)	69 (13.4)	18 (16.4)	47 (10.3)	48 (14.4)
**UHDRS motor**	34 (0-106	31 (0-105)	29 (2-94)	37.5 (2-106)	35 (0-101)
**TFC**	8 (0-13)	8.2 (0-13)	8.1 (0-13)	7.5 (0-13)	7.4 (0-13)
**Stage 1 (%)**	389 (30.4)	166 (37.3)	25 (27.2)	130 (31.6)	58 (20.3)
**Stage 2 (%)**	405 (31.6)	136 (30.6)	37 (40.2)	100 (24.3)	119 (41.6)
**Stage 3 (%)**	333 (26)	94 (21.1)	24 (26.1)	118 (28.7)	80 (28)
**Stage 4/5 (%)**	154 (12)	49 (11)	6 (6.5)	63 (15.3)	29 (10.1)

**Table 2. **Demographic, clinical and genetic data from REGISTRY participants. All participants (apart from Polish) with core data set (age, CAG repeat larger allele, UHDRS motor score, TFC). ^1^ Germany, Austria, Switzerland, Netherlands, Belgium; ^2^ Denmark, Norway, Finland; ^3^ Italy, Spain, Portugal. CAG repeat information was from BioRep when both local and BioRep were available. Demographic and CAG repeat information is from all participants with core data (Central European n=514; Nordic n=110; Southern European n=457; UK n=286). TFC, disease stage and UHDRS motor score are from all manifest participants (Central European n=445; Nordic n=92; Southern European n=410; UK n=286).

Investigators estimated that 615 (48%) participants with manifest HD had motor signs at onset while 251 (19.6%) had a psychiatric onset, 107 (8.4%) first had cognitive signs and 169 (13.2%) had a mixed onset. Information was missing from 125 (9.8%). 503 participants (39.3%) had a life-time history of severe psychiatric signs (psychosis, aggression and suicidal ideation) - 126 (9.8%) participants had two or more severe psychiatric signs and 21 participants (1.6%) had all three. 151 (11.8%) had a life-time history of delusions and/or hallucinations, 244 (19.1%) had disruptive or aggressive behaviour and 255 (19.9%) had suicidal ideation or suicide attempts with a total of 90 recorded suicide attempts in all stages of HD (stage 1: 14 (15.6%); stage 2: 27 (30%); stage 3: 29 (32.2%); stage 4/5: 20 (22.2%)). A high mood subscore of the behavioural score was highly predictive of a suicide attempt (logistic regression analysis, z-test=3.84, p=0.0001) whereas motor, cognitive or the behavioural subscores for apathy, compulsions, psychosis or irritability were not.  

The majority of participants who underwent behavioural assessment had behavioural abnormalities (depression, apathy, irritability) in addition to motor signs (694 (86.6%) while 107 (13.4%) had none.  

*Co-morbid conditions and medication*

A co-morbid condition, interfering morbidity or previous intervention was recorded in 706 participants (40.0%). Most common were essential primary hypertension (ICD-10 code I10, 133 participants), pure hyper-cholesterolaemia (E78.0. 62) asthma (J45, 48), diabetes mellitus (E10-E14, 26), hypertrophy of prostate (N40, 27), unspecified arthritis (M13.9, 25) and unspecified hypothyroidism (E03.9, 24). Co-morbid conditions were then grouped revealing that 67 participants (3.8%) had ‘neoplasms’ (ICD-10 C and D), 187 (10.6%) had ‘endocrine disorders’ (ICD 10 E), 227 had ‘cardiovascular disorders’ (ICD-10 G and I), and 148 suffered physical ‘trauma’ as interfering morbidity (ICD-10 S). ‘Trauma’ was common across all stages but was less frequent in stage 4/5 (stage 1: 50 (33.8%), stage 2: 43 (29.1%), stage 3: 30 (20.3%), stage 4/5: 12 (8.1%)).

Most participants were taking medication (1022, 57.9%). The most commonly taken medications are listed in Table 3 with their respective medication class in Table 4.

**Table 3**. The most commonly prescribed drugs

**Table:** 

**Medication**	**ATC code**	**n**	**Typical indications**
Tiapride hydrochloride	N05AL03	148	Chorea/hyperkinesias
Olanzapine	N05AH03	133	Chorea/dyskinesia/aggression/psychosis
Risperidone	N05AX08	121	Chorea/Dyskinesia/aggression/psychosis
Citalopram hydrobromide	N06AB04	120	Depression/irritability
Paroxetine hydrochloride	N06AB05	120	Depression/irritability
Haloperidol	N05AD01	109	Chorea
Clonazepam	N03AE01	79	Anxiety
Amantadine hydrochloride	N04BB01	76	Chorea/dyskinesia
Mirtazapine	N06AX11	75	Depression/insomnia
Tetrabenazine	N07XX06	69	Chorea/dyskinesia
Lorazepam	N05BA06	54	Anxiety
Sulpiride	N05AL01	46	Chorea/dyskinesia/irritability

**Table 3.** The most commonly prescribed drugs. A total of 3074 concomitant medications were recorded. ATC: Anatomical Therapeutic Chemical

**Table 4. **Medication classes

**Table:** 

**Medication class**	**All manifest participants**	**Central European**	**Northern European**	**Southern European**	**UK**	**Poland**
Anti-dyskinetic	607 (39.3%)	199 (37.2%)	18 (18%)	263 (56%)	94 (23.9%)	33 (70%)
Anti-depressant	702 (45.6%)	203 (38.1%)	39 (39%)	270 (57.7%)	169 (43.1%)	21 (45.7%)
Anti-dementia	65 (4.2%)	45 (8.4%)	6 (6%)	12 (2.6%)	0	2 (4.3%)
Nutritional supplements	171 (11.1%)	78 (14.4%)	12 (12%)	58 (12.4%)	16 (4.1%)	7 (14.9%)
Other	335 (21.8%)	142 (26.6%)	19 (19%)	76 (16.2%)	79 (20.2%)	19 (40.4%)

**Table 4.** Medication classes. We defined 5 groups of medication. Medications to treat motor signs (‘anti-dyskinetics’) comprised anti-psychotics (N05AA01), tetrabenazine (N07XX06) and anti-parkinsonian medications (N04A, N04B); ‘anti-depressants’ comprised medications coded as anti-depressants (N06A, N06C), anxiolytics (N05B, N05C), lithium (N05AN01), and anti-epileptics (N03A) where the indication was ‘depression’ or ‘mood stabilisation’. ‘Anti-dementia’ included medications coded as anti-dementia (N06D) or psychostimulants (N06B). ‘Nutritional supplements’ were all medications with ATC codes A11, B03BB01, C10AX06, H05BA01, N06BX13, or C01EB05. All other medications were combined as ‘others’. In manifest participants, ‘anti-dyskinetics’ were used more frequently in Southern Europe and Poland (p<0.0001), and in Southern Europeans ‘anti-depressants’ were used somewhat more frequently than in other regions (p<0.0001). Use of all other medications was similar across regions. ATC: Anatomical Therapeutic Chemical

**

*Genotype and phenotype across European regions*

Across contributing European regions gender distribution was similar (Table 2; χ^2^_4_ = 2.5273, n.s.). CAG repeat length differed statistically between regions (p<0.05 and p<0.0001, respectively); participants from Northern Europe had slightly shorter repeat lengths than those from Central Europe (p<0.0001), Southern Europe (p<0.0001) and the UK (p=0.0001, Table 2) probably because there were no Nordic participants with very large CAG repeat length expansions.; Southern European participant’s CAG repeat lengths were slightly larger than those of participants from Central Europe (p<0.01) or from the UK (p<0.01). Central European participants were younger than participants from Southern Europe (p=0.01) and the UK (p<0.05), and UHDRS motor scores were also similar apart from Southern European participants who had a slightly higher motor score than Central Europeans (p<0.0001), Northern Europeans (p<0.01) and the UK (p<0.05), with no further differences observed between other regions (Table 2). Central Europeans were slightly less advanced in HD than Southern Europeans (p=0.01) and UK participants (p<0.01).

*Association of phenotype with disease burden*

We related the severity of the clinical signs across three domains of HD (motor, behaviour (n=801), cognition (n=676)) to a measure of the biological disease burden calculated from a participant’s age and CAG repeat length ((CAG_n_-35.5) X age = disease burden: [20]. UHDRS motor score increased in severity with increasing disease burden (linear regression, adjusted R^2^=0.19, p<0.0001, Figure 1A) while cognitive composite score (UHDRS total correct for letter fluency, symbol digit modalities test and Stroop subscores for word reading, colour naming and interference; linear regression, adjusted R^2^=0.1224, p<0.0001 Figure 1B) or equally weighted scores (adjusted R^2^=0.1245, p<0.0001) and function declined (linear regression, adjusted R^2^=0.1172, p<0.0001, Figure 1C). In contrast, there was no linear association of the total behavioural score, or the subscores mood (depression, low self esteem, anxiety, freq*severity), psychosis (delusions and hallucinations, freq*severity), irritability/aggression (disruption, aggression and perseverations, freq*severity) or compulsions (freq*severity) with disease burden (Figure 1D). Only apathy (freq*severity) was very weakly associated with disease burden (adjusted R^2^=0.01, p=0.0024).

**Figure 1**

**Figure:**
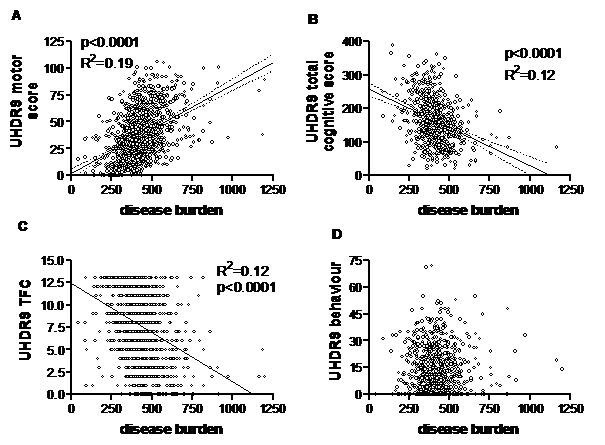

**Figure 1. **Association of UHDRS domain score, or TFC, with disease burden. **A.** Linear increase of motor score with increasing disease burden. **B.** Linear decrease of cognitive score with increasing disease burden. **C.** Linear decline of TFC with increasing disease burden. **D.** No linear association of behavioural score with disease burden.

*The variability of the phenotype*

The amount of variability of the scores in the three symptom domains of HD explained by disease burden alone was low. We therefore assessed the extent to which biological (‘age’, ‘CAG repeat length’) or environmental factors (‘region’, ‘medication’, ‘co-morbidity’) contributed to this variability. Overall, 23% of the variation of the motor score was explained by the factors in the final multiple linear regression model (p<0.0001, adjusted R^2^=0.23). A higher motor score was associated with the use of ‘anti-dyskinetics’, with the presence of ‘trauma’, with increasing age and increasing CAG repeat lengths (Figure 2). For the behavioural score, 12% of the variation of the behavioural score was explained by the factors in the final model (p<0.0001, adjusted R^2^=0.12). The use of ‘anti-depressants’ and ‘anti-dyskinetics’, and the presence of endocrine co-morbidity, was associated with a higher behavioural score. Overall, 24% of the variation of the composite cognitive score was explained by the factors in the final model (p<0.0001, adjusted R^2^=0.24), and 25% of the cognitive score where items were weighted equally (p<0.0001, R^2^=0.25). A higher cognitive score was associated with the use of ‘nutritional supplements’ and ‘other medications’ whereas a lower score was associated with the use of ‘anti-dyskinetic’ medication, increasing CAG repeats and age. 

**Figure 2**

**Figure:**
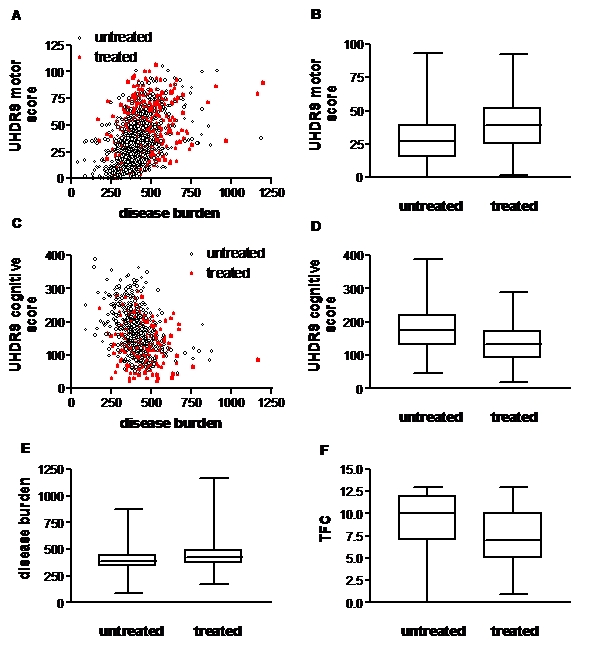

**Figure 2**. Treatment with anti-dyskinetic medications. Participants on medication for motor signs (‘treated’ with anti-dyskinetic, dopamine depleting or dopaminergic) had a higher motor score than un-treated participants (Mann-Whitney U test, p<0.0001, **A** and **B**), a lower cognitive score (p<0.0001, **C** and **D**) and a lower TFC (p<0.0001, **F**). The disease burden score was also different (p<0.0001, **E**). However, the difference between treated and un-treated participants was far less.

*Total functional capacity*

Finally, we investigated whether changes in the scores in the three domains, biological (‘age’, ‘CAG repeat length’) or environmental factors (‘region’, ‘medication’, ‘co-morbidity’) predicted disease stage. To this end we used multi-nomial logistic regression analysis with the disease stage as dependent variable and domain scores, biological and environmental factors as independent variables. For cognitive scores we used the composite score, an equally weighted score or the subscores. In the final model, the factors ‘motor score’, ‘region’ and ‘cognitive score’ explained 28% of the variability of disease stage. The results were similar when using the composite cognitive score or an equally weighted cognitive score, or the symbol digit test as single subscore. Internal validation using the same data correctly classified about 59% of all participants across all disease stages compared with the actual disease stages (table 5). The ROC analysis of the final model accurately classified more than 80% for each individual stage except for disease stage 2 with an accuracy of about 75% (Table 5 and Figure 3).  

**Table 5**. Comparing the accuracy of multinomial logistic regression analyses with different types of cognitive score

**Table:** 

** **	** **	**Accuracy**			
**Cognitive score**	**Proportion correct (%)**	**Disease Stage 1**	**Disease Stage 2**	**Disease Stage 3**	**Disease Stage 4/5**
Cognitive score	58.9	84.8	73.9	82.6	86.2
Cognitive equally weighted	59.5	84.8	74.1	82.4	86.5
Subscores (symboltest)	58.5	84.5	74.2	81.9	83.1

  **Table 5**. Comparison of the performance of different multinomial logistic regression models with the total sum cognitive score, equally weighted cognitive score or symbol digit subscore.

**Figure 3**

**Figure:**
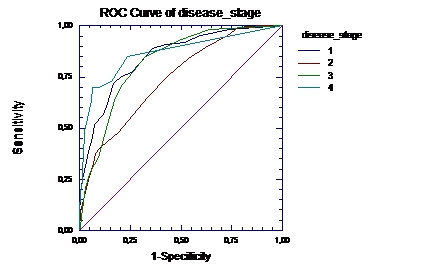

**Figure 3.** Receiver operating characteristic (ROC) curves plot the sensitivity versus (1-specificity) for classifying each disease stage (stages 1-4/5).

**Discussion**

We report on the first cross-sectional data cut of REGISTRY, EHDN’s large scale multi-centre multi-national prospective observational study of HD. 

The proportions of gene mutation carriers, manifest participants, and among these juvenile and late-onset participants, were similar across regions. Age and gender distribution, CAG repeat length, and the severity of the core symptom domains of HD (motor, cognitive, behaviour) as well as functional capacity were broadly comparable across different European regions. Ethnicity may influence the prevalence of HD; in Japanese for instance, and possibly in Sub-Saharan Africans, HD may be much rarer than in Caucasian populations [21]. Whenever HD manifests, however, the phenotype and progression of the disease appear similar. This is in agreement with data from Venezuela where the phenotype and CAG repeat length expansions are similar to participants from North America or Europe [22] . Europeans taking part in this study were mainly Caucasian. Despite sharing much of their genetic makeup, extensive SNP maps suggest that Northern and Southern European populations differ to some extent [23] . This suggests that the CAG repeat expansion in the *HTT* gene influences the phenotype in a similar way across Europe. However, environmental influences may differ, one example being medication with more than half of all manifest participants taking medication. Investigators prescribed similar medication classes. Southern European and Polish investigators, however, used anti-dyskinetic medication more frequently than investigators in other regions. 

Collapsing contributing countries into regions was somewhat arbitrary. In the future, with larger numbers of participants we could re-examine whether extracting any one country from a regional bin leads to a different finding. Any difference could reflect different genetic, or environmental, influences within that given country. However, the similar CAG repeat genotypes and phenotypes across European regions imply that one can conduct treatment trials across Europe without the need for stratification according to country. Considering the potential scale of these trials, this is encouraging.  

*The evolution of the phenotype, and its variability*

Since the phenotype was similar in European regions we evaluated the evolution of HD in the whole cohort of participants. Good evidence suggests that biological factors, in particular CAG repeat length and age, contribute to when first symptoms manifest and how HD evolves biologically [20] [22] [24] [25] . Thus we related the evolution in three clinical domains, i.e. motor, cognitive and behaviour, to the continuous measure of biological disease burden that is linearly associated with the neuropathological disease stage and is closely related to striatal volumes [20]. In contrast to clinical ratings of disease evolution, such as TFC, disease burden consists of unambiguous variables. Our data indicate that motor score and TFC increased and cognitive score decreased monotonically with an increasing biological disease burden. This is in accord with longitudinal observations of clinical evolution alone [26]. In contrast, with the exception of the apathy subscore, behavioural scores were not associated with disease burden. The effective treatment of many behavioural problems, e.g. depression, irritability, aggression or psychosis may suggest these behaviours are episodic rather than progressive. If left untreated, however, it is possible that symptoms such as e.g. irritability may progress, and it is likely that some behaviours do reflect degenerative neuropathological processes. The treatment of apathy proves much more difficult; sometimes apathy may improve with successful treatment of a depressive episode or psychosis suggesting it may be part of several different underlying psychopathologies including depression and, as a negative syndrome, psychosis [27] [28] . Current concepts of what constitutes apathy, and how to diagnose it, have recently been reappraised [27] [28] . Since apathy is currently not well defined, and its origins may be quite different, it is perhaps not surprising that the association of apathy with disease burden is not as strong as that of motor or cognitive signs.

Taken together, these data suggest that the clinical evolution of HD, and the subsequent impediments in daily life, reflects the neuropathological changes accruing over time. The relation of clinical findings to biological factors extends previous descriptions of the clinical evolution of HD [26]. However, overall, the variability of the clinical scores explained by the disease burden score was moderate. One explanation might be that CAG repeat length and age are only two of many biological factors determining the evolution of HD [22] . Environmental factors, e.g. medication, may also contribute to the variability. The use of anti-dyskinetic medication for instance influenced all three domains since it was associated with a higher motor score, a higher behavioural score and a lower cognitive score. 

The strength of our study is the large number of participants with data collected across Europe following the same study protocol. We demonstrate that such studies can be conducted effectively across different countries and multiple languages. In addition, investigators are regularly trained and certified to improve data quality. REGISTRY, unlike many observational clinical research initiatives, engaged data monitoring based on the principles of ICH-GCP. Participating sites are visited regularly by a team of trained monitors in order to ensure the plausibility and accuracy of the data, and to promote adherence to the study protocol and its procedures. Data monitoring is not a prerequisite for cohort studies, but it is an investment to enhance the collection of more robust and reliable data. 

All predictions on the evolution of the HD phenotype from the present cross-sectional analyses need to be tested further in an appropriate longitudinal follow-up design that examines relative effects over time. The REGISTRY database continues to expand and should allow such studies in the very near future. In addition, given more time and repeated visits, missing data can be resolved such that both the proportion and the total number of complete data sets increases. This includes the length of time and dosing regime of participants on medication, which were not taken into account in the present study. 

A separate concern relates to whether the sample is truly representative. The database necessarily reflects the HD population who live near to neurological HD services, which are predominantly located in cities. The present cohort may also have been biased towards excluding individuals with active, e.g. psychiatric problems leaving less time to participate in the study. Late-stage participants were also under-represented illustrating their difficulties in attending out-patient clinic and the limitations using currently available scales. 

Many studies of HD, including REGISTRY, rely on assessment scales that depend on subjective impressions of clinicial ratings. Rating scales themselves may also differ in quality and thus introduce data variability. The UHDRS has not (yet) been evaluated using analytical methods such as the Rasch analysis or Item Response Theory [29], although such analyses are well under way. However, the variability of clinical signs and their ratings also underlines the need to identify and validate better biomarkers of disease progression.   

The unparalleled large collection of clinical data and biomaterials in REGISTRY will enable research projects to be conducted on a scale that has not previously been possible. The initiative will expedite the search for disease modifiers (genetic and environmental) of age at onset and disease progression that could be harnessed for the development of novel treatments, thus offering a promising new direction towards slowing down or preventing this debilitating disease. 

**Acknowledgements**

We wish to acknowledge the time and effort of all participants in this study. EHDN is supported and funded by the CHDI Foundation Inc.. We thank Marc Sutton for invaluable help with converting medication entries into the ATC code.

**Funding information**

The European Huntington’s Disease Network is funded by CHDI Foundation, Inc.

**Competing interests**

The authors have declared that no competing interests exist.

**Writing committee:** M Orth, OJ Handley, C Schwenke, SB Dunnett, D Craufurd, A Ho, EJ Wild, SJ Tabrizi, GB Landwehrmeyer

**Registry Steering committee:**  A-C Bachoud-Lévi, AR Bentivoglio, I Biunno, R Bonelli, J-M Burgunder, SB Dunnett, JJ Ferreira, OJ Handley, A Heiberg, T Illmann, GB Landwehrmeyer, J Levey, JE Nielsen, M Päivärinta, RAC Roos, A Rojo Sebastián, SJ Tabrizi, W Vandenberghe, C Verellen-Dumoulin, J Zaremba, T Uhrova, J Wahlström

**Biostatistics:** C Schwenke, M Orth

**Information technology:** T Illmann, M Wallner

**Language coordinators:** K Barth, M Bascuñana Garde, R Bos, D Ecker, OJ Handley, N Heinonen, C Held, M Laurà, A Martínez Descals, T Mestre, D Monza, J Naji,  M Orth, H Padieu, S Pro Koivisto, A Rialland, P Sasinková,  P Trigo Cubillo, M van Walsem, M-N Witjes-Ané, D Zielonka

**Contributors:**

**AUSTRIA:**

**Graz (LKH Graz, Abteilung für Psychiatrie):** Raphael M. Bonelli; Brigitte Herranhof;  Anna Hödl; Michael Koppitz; Markus Magnet; Daniela Otti; Annamaria Painold; Karin Reisinger

**BELGIUM**:

**Brussels (VUB, Neurology): **Anja Flamez; Vera Morez; Sylvie de Raedt

**Charleroi (Institut de Pathologie et de Génétique (IPG)):** Pascale Ribaï; Christine Verellen-Dumoulin

**Leuven (Universitair Ziekenhuis Gasthuisberg):** Wim Vandenberghe; Dimphna van Reijen

**DENMARK:**

**Copenhagen (Hukommelsesklinikken, Rigshospitalet):**  Lis Hasholt; Lena E. Hjermind; Oda Jakobsen; Anne Nørremølle; Sven Asger Sørensen; Jette Stokholm

**FINLAND**:

**Helsinki-Vaestoliito (Department of Medical Genetics  Väestöliitto):** Maarit Peippo; Marjatta  Sipponen

**Turku-Suvituuli (Rehabilitation Centre Suvituuli):**  Heli Hiivola; Kirsti Martikainen; Katri Tuuha

**GERMANY:**

**Aachen (Universitätsklinikum Aachen, Neurologische Klinik):** Christoph Michael Kosinski; Daniela Probst; Christian Sass; Johannes  Schiefer; Christiane Schlangen; Cornelius J. Werner

**Berlin (Klinik und Poliklinik für Neurologie - Charité - Universitätsmedizin Berlin):** Josef Priller; Harald Prüß

**Bochum (Huntington-Zentrum (NRW) Bochum im St. Josef-Hospital):** Jürgen Andrich;  Rainer Hoffmann; Peter Kraus; Christian Prehn; Carsten Saft; Stephan Salmen; Katrin Straßburger

**Dinslaken (Gesundheitszentrums Lang in Dinslaken):** Herwig Lange

**Dresden (Universitätsklinikum Carl Gustav Carus an der Technischen Universität Dresden, Klinik und Poliklinik für Neurologie):** Ulrike Hunger; Matthias Löhle; Simone Schmidt; Alexander Storch; Anett Wolz; Martin Wolz

**Freiburg (Universitätsklinik Freiburg, Neurologie):** Johann Lammbeck; Birgit Zucker

**Hamburg (Universitätsklinikum Hamburg-Eppendorf, Klinik und Poliklinik für Neurologie):** Ute Hidding; Alexander Münchau; Michael Orth; Lars Stubbe

**Heiligenhafen (Psychatrium Heiligenhafen):** Walburgis Heinicke; Michael Orth

**Marburg KPP (Klinik für Psychiatrie und Psychotherapie Marburg-Süd):** Bernhard Longinus

**Marburg Uni (Universität Marburg, Neurologie):** Jens Carsten Möller; Ida Rissling

**München (Huntington-Ambulanz im Neuro-Kopfzentrum - Klinikum rechts der Isar der Neurologischen Klinik und Poliklinik der Technischen Universität München):** Alexander Peinemann; Michael Städtler; Adolf Weindl

**Münster (Universitätsklinikum Münster, Klinik und Poliklinik für Neurologie):** Stefan Bohlen; Herwig Lange; Ralf Reilmann

**Taufkirchen (Isar-Amper-Klinikum - Klinik Taufkirchen (Vils)):** Antonie Beister; Matthias Dose; Gabriele Leythaeuser; Ralf Marquard; Caroline Schrenk; Michele Schuierer;  Alexandra Wiedemann

**Ulm (Universitätsklinikum Ulm, Neurologie):** Daniel Ecker; Bernhard Landwehrmeyer; Franziska Lezius; Sonja Trautmann

**ITALY:**

**Florence (Neurologia I- Unita' di Neurogenetica  Departimento di Neurologia e Psichiatria, Universita' di Firenze):** Elisabetta Bertini; Claudia Mechi; Marco Paganini; Sivia Piacentini; Maria Romoli; Sandro Sorbi

**Genoa (Dipartimento di Neuroscienze, Oftalmologia e Genetica (DiNOG)  Università di Genova):** Giovanni Abbruzzese; Monica Bandettini di Poggio; Emilio Di Maria; Giovanna Ferrandes; Paola Mandich; Roberta Marchese

**Milan **(**Fondazione IRCCS Istituto Neurologico C. Besta, Milan):** Alberto Albanese; Stefano Di Donato; Caterina Mariotti; Paola Soliveri

**Naples (Azienda Ospedaliera Universitaria Federico II - Dipartimento di Scienze Neurologiche):** Rinaldi Carlo; Di Maio Luigi; Giuseppe De Michele; Carlo Rinaldi; Elena Salvatore; Tecla Tucci

**Pozzilli (Neuromed, PARCO TECNOLOGICO (Centro Studi)):** Andrea Ciarmiello; Tiziana Martino; Maria Simonelli; Ferdinando Squitieri

**Rome (Istituto di Neurobiologia e Medicina Molecolare CNR/ Istituto di Neurologia, Dipartimento di Neuroscienze/ CNR Istituto di Scienze e Tecnologie della Cognizione):** Anna Rita Bentivoglio; Alfonso Fasano; Marina Frontali; Arianna Guidubaldi; Tamara Ialongo; Gioia Jacopini; Giovanna Loria; Carla Piano; Silvia Romano; Francesco Soleti; Maria Spadaro; Paola Zinzi

**NORWAY**

**Oslo-RH (Rikshospitalet, Dept. of Medical Genetics):**  Arvid Heiberg; Marleen R van Walsem

**Oslo-Ulleval:**  Kathrine Bjørgo; Madelein Fannemel; Per Gørvell. Lars Retterstøl

**Trondheim (St. Olavs Hospital):** Inga Bjørnevoll; Sigrid Botne Sando

**POLAND**

**Gdansk (St Adalbert Specialistic Hospital, Gdansk Zaspa):** Emilia Jadwiga Sitek; Jaroslaw Slawek; Witold Soltan

**Katowice (Silesian Medical University Katowice): **Magdalena Boczarska-Jedynak, Barbara Jasinska-Myga, Gregorz Opala

**Krakow (Krakowska Akademia Neurologii):** Monika Rudzińska; Andrzej Szczudlik; Magdalena Wójcik, Krzysztof Banaszkiewicz

**Poznan (Medical University of Poznań): **Anna Bryl; Anna Ciesielska; Aneta Klimberg;  Wojciech Kozubski; Jerzy Marcinkowski; Pani Justyna Sempołowicz; Daniel Zielonka

**Warsaw-MU (Medical University of Warsaw, Neurology):** Piotr Janik; Anna Kalbarczyk; Hubert Kwiecinski; Zygmunt Jamrozik

**Warsaw-IPiN (Institute of Psychiatry and Neurology  Dep. of Genetics, Dep. of Neurology):** Jakub Antczak; Grzegorz Witkowski, Maryla Rakowicz;Przemyslaw  Richter; Danuta Ryglewicz; Jacek Zaremba; Elzbieta Zdzienicka

**PORTUGAL**

**Lisbon-Fernando Fonseca (Hospital Fernando Fonseca, Serviço de Neurologia):** Christina Costa

**Lisbon-Santa Maria (Neurological Clinical Research Unit, Institute of Molecular Medicine**: Miguel Coelho; Joaquim J Ferreira; Tiago Mestre; Mário M Rosa; Anabela Valadas

**Porto-São João (** **Hospital São João E.P.E.):** Miguel Gago; Carolina Garrett; Maria Rosalia Guerra

**SPAIN**

**Barcelona-Bellvitge (Hospital Universitari de Bellvitge): **Jordi Bas; Matilde Calopa

**Barcelona (Hospital Mútua de Terrassa) :** Miquel Aguilar Barberà; Dolores Badenes; Laura Casas; Sonia Escalante Arroyo; Jorge Hernández Vara; Jerzy Krupinski; Judith López; Marta Obdulia; Pilar Quilez Ferrer; Ana Rojo Sebastián; Silvia Romero Contreras; Gemma Tome Carruesco

**Burgos (Servicio de Neurología Hospital General Yagüe):** Esther Cubo; Natividad Mariscal; Jesús Sánchez

**Granada:** Francisco J Barrero (Hospital Universitario San Cecilio, Neurología); Blas Morales & José Luis López-Sendón Moreno (Hospital Universitario Ramón y Cajal, Neurología)

**Madrid-Clinico (Hospital Clínico Universitario San Carlos):** Rocío García-Ramos García; Purificacion Pin Quiroga; Clara Villanueva

**Madrid FJD: (Madrid-Fundación Jiménez Díaz):** Pedro-José García Ruíz-Espiga; Asunción Martínez; María José Saiz Artiga; Vicenta Sánchez

**Madrid RYC (Hospital Ramón y Cajal, Neurología):** Mónica Bascuñana; Marta Fatas; Guillermo García Ribas; Justo García de Yébenes; José Luis López Moreno; Christine Schwarz; Patricia Trigo Cubillo

**Palma (Hospital Son Dureta):** Penelope Navas Arques; Aranzazú Gorospe; Inés Legarda; María José Torres Rodríguez

**Pamplona (Hospital Virgen del Camino, Medical Genetic):** Itziar Gaston; Maria A. Ramos-Arroyo

**Zaragoza (Hospital Clinico Universitario “Lozano Blesa” de Zaragoza):** Javier López del Val; Laura Martinez

**SWITZERLAND**

**Bern:** Jean-Marc Burgunder (Neurologische Klinik des Inselspitals); Irene Romero; Michael Schüpbach; Sabine Weber Zaugg (Zentrum für Bewegungsstörungen, Neurologische Klinik und Poliklinik)

**THE NETHERLANDS**

**Enschede (Medisch Spectrum Twente):** Monique S.E. van Hout; Jeroen P.P. van Vugt; A. Marit de Weert

**Groningen (Polikliniek Neurologie):**  J.J.W. Bolwijn; Meike Dekker; K.L. Leenders; Joost.C.H. van Oostrom

**Leiden (Leiden University Medical Centre (LUMC)):** Reineke Bos; Eve Dumas; Caroline K. Jurgens; Raymund A.C. Roos; Marie-Noëlle Witjes-Ané

**U.K.**

**Aberdeen (NHS Grampian, Clinical Genetics Centre):** Kirsty Matheson; Daniela Rae; Sheila Simpson; Fiona Summers; Alexandra Ure

**Birmingham** **(The Barberry Centre, Dept of Psychiatry):** Adrienne Curtis; Jenny Keylock; Hugh Rickards; Jan Wright

**Cambridge (Cambridge Centre for Brain Repair, Forvie Site):** Roger A. Barker; Kate Fisher; Anna Olivia Goyder Goodman; Susan Hill; Ann Kershaw; Sarah Mason; Nicole Paterson; Lucy Raymond

**Cardiff (The Institute of Medical Genetics, University Hospital of Wales):** Jon Bisson; Monica Busse;  Lynda Ellison-Rose; Olivia Handley; SB Dunnett; Jenny Naji; Kathy Price; Anne Rosser

**Edinburgh (Molecular Medicine Centre, Western General Hospital, Department of Clinical Genetics)**: Maureen Edwards; Paul A. De Sousa; Teresa Hughes; Marie McGill; Pauline Pearson; Mary Porteous; Adam Zema

**Fife (Scottish Huntington's Association Whyteman's Brae Hospital)**: Peter Brockie; Jillian Foster; Nicola Johns; Sue McKenzie; Gareth Thomas

**Gloucester** **(Department of Neurology Gloucestershire Royal Hospital)**: Liz Burrows; Amy Fletcher; Fiona Laver; Mark Silva; Aileen Thomson

**Leeds (Chapel Allerton Hospital, Department of Clinical Genetics):** Carol Chu; Emma Hobson; Stuart Jamieson; Jean Toscano; Sue Wild; Pam Yardumian

**Leicester (Leicestershire Partnership Trust, Mill Lodge):** Colin Bourne; Carole Clayton; Heather Dipple; Janet Grant; Diana Gross; Caroline Hallam; Julia Middleton; Ann Murch

**London (Guy's Hospital):** Thomasin Andrews; Andrew Dougherty; Fred Kavalier; Charlotte Golding; Alison Lashwood; Dene Robertson; Deborah Ruddy; Anna Whaite

**London (The National Hospital for Neurology and Neurosurgery):** Thomasin Andrews;  Stefania Bruno; Charlotte Golding; Susie Henley; Marianne Novak; Christine O'Driscoll; Aakta Patel; Elisabeth Rosser; Sarah Tabrizi; Rachel Taylor; Thomas Warner; Edward Wild

**Manchester (** **Genetic Medicine, University of Manchester, Manchester Academic Health Sciences Centre and Central Manchester University Hospitals NHS Foundation Trust):** Natalie Arran; David Craufurd; Ruth Fullam; Liz Howard; Susan Huson; Lucy Partington-Jones; Nichola Ritchie; Julie Snowden; Annie Solom; Cheryl Stopford; Jennifer Thompson; Leann Westmoreland

**Oxford** **(Churchill Hospital):** Andrea H Nemeth; Gill Siuda

**Sheffield (The Royal Hallamshire Hospital):** Oliver Bandmann; Alyson Bradbury; Kay Fillingham; Isabella Foustanos; Oliver Quarrell; Hazel Reynders; Lisa Robertson; Katharine Tidswell
